# *N*-(2,3,5,6-Tetrafluoropyridin-4-yl)formamide

**DOI:** 10.1107/S2414314622008045

**Published:** 2022-08-16

**Authors:** Bailey D. Newell, Colin D. McMillen, John P. Lee

**Affiliations:** a The University of Tennessee at Chattanooga, Department of Chemistry and Physics, #2252, 615 McCallie Avenue, Chattanooga, TN 37403, USA; b Clemson University, Department of Chemistry, Clemson, SC 29634, USA; University of Aberdeen, Scotland

**Keywords:** crystal structure, amide bond, perfluorinated pyridine, formamide, hydrogen bonding

## Abstract

The title compound, C_6_H_2_F_4_N_2_O, displays amide bond lengths of 1.218 (3) Å and 1.366 (3) Å for the C=O and C—N bonds, respectively. The C_p_—N—C—O (p = pyridine) torsion angle of 179.0 (2)° indicates an anti-conformation for the grouping. Inter­molecular hydrogen bonding is observed between the amine N—H group and the carbonyl O atom, which generates chains of mol­ecules propagating along the *b-*axis direction.

## Abstract 2

In the title compound, the C_p_—N—C—O (p = pyridine) grouping has an anti conformation.

## Graphical abstract

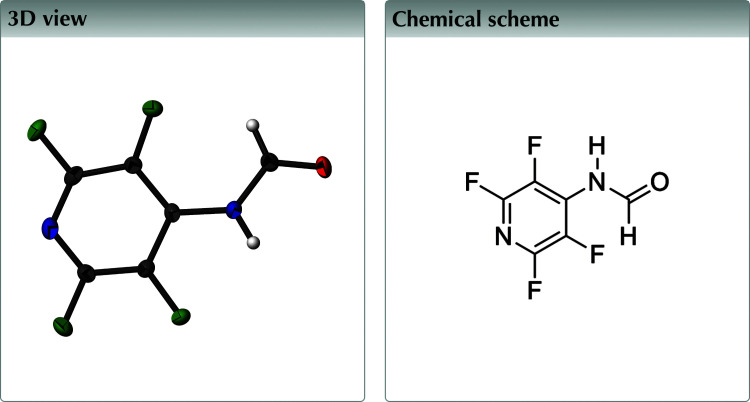

## Structure description

The title compound, *N*-(2,3,5,6-tetrafluoropyridin-4-yl)formamide, (I), contains a perfluorinated pyridine heterocycle and a formamide group *para* to the pyridine N atom. These groups have shown utility in independent biochemical applications. For example, fluoro­aromatic compounds are used in positron emission tomography (Hashizume *et al.*, 1996 ▸) and pyridine rings can act as the bioisosteres of amides (Sun *et al.*, 2019 ▸). The structure reported here combines these components and could be of inter­est for biochemical applications. A search of the CCDC shows no structures that contain a pyridine ring functionalized with a formamide group in the 4-position (Groom *et al.*, 2016 ▸).

**Figure 1:**
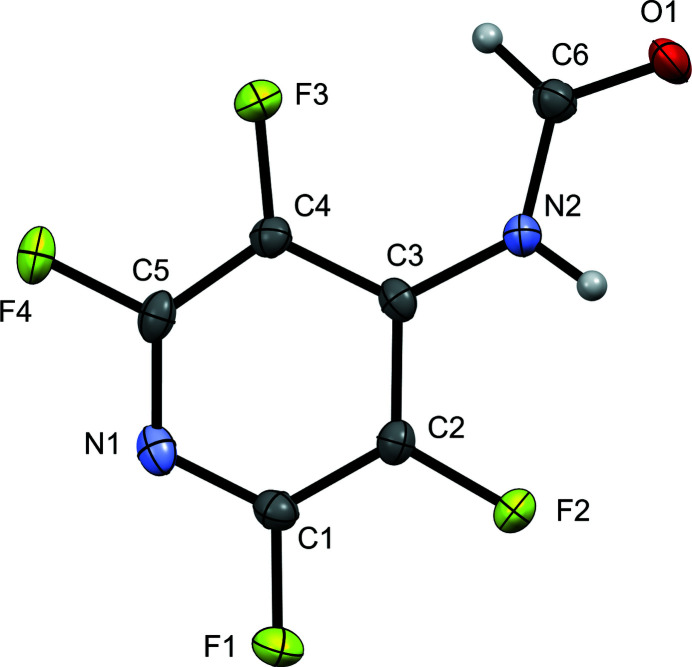
Displacement ellipsoid perspective view (50% probability) for the title structure showing the atom-numbering scheme.

The crystal structure of (I) represents the first example of a perfluorinated pyridine ring with a formamide functional group (Fig. 1 ▸). The amide bond lengths for (I) are 1.218 (3) Å and 1.366 (3) Å for the C=O and C—N bonds, respectively, which are in good agreement with the corresponding bonds in the related compounds *N*-phenyl­formamide (Omondi *et al.*, 2014 ▸), *N*-(2,6-di­fluoro­phen­yl)formamide (Omondi *et al.*, 2009*b* ▸), and *N*-(2,6-di­bromo­phen­yl)formamide (Omondi *et al.*, 2009*a* ▸). As observed in other structures, the amide bond lengths for (I) are consistent with little to no N=C/C—O bond resonance contribution. The formamide group and pyridine ring in (I) are somewhat twisted with a dihedral angle of 13.21 (5)°. This is similar to *N*-phenyl­formamide (Omondi *et al.*, 2014 ▸) (dihedral angle between the benzene ring and formamide groups = 10.5°) but in contrast to *N*-(2,6-di­fluoro­phen­yl)formamide (Omondi *et al.*, 2009*b* ▸) and *N*-(2,6-di­bromo­phen­yl)formamide (Omondi *et al.*, 2009*a* ▸) where the equivalent dihedral angles are 58.4 and 83.2°, respectively. The latter structures indicate that the steric effects likely increase from H to F to Br; however, this large deviation from planarity is not observed in (I). Furthermore, the torsion angle of 179.0 (2)° for C3—N2—C6—O1 in (I) indicates a near *anti*-conformation, but in structures with a benzene ring, the carbon­yl–benzene conformation is *syn* regardless of aromatic substituents (Omondi *et al.*, 2009*a* ▸,*b* ▸, 2014 ▸). Taken together, these indicate that the pyridine ring is playing a role in the structure beyond the sterics of the aromatic ring substituents. The pyridyl related compounds *N*-(3,5-di­chloro-2-pyrid­yl)formamide (Resinger *et al.*, 2005 ▸) and form­yl(2-pyrid­yl)amine (Bock *et al.*, 1996 ▸) also show an *anti*-conformation for the carbonyl and pyridine ring as well as near coplanarity of the functional groups as observed for the title compound.

**Figure 2:**
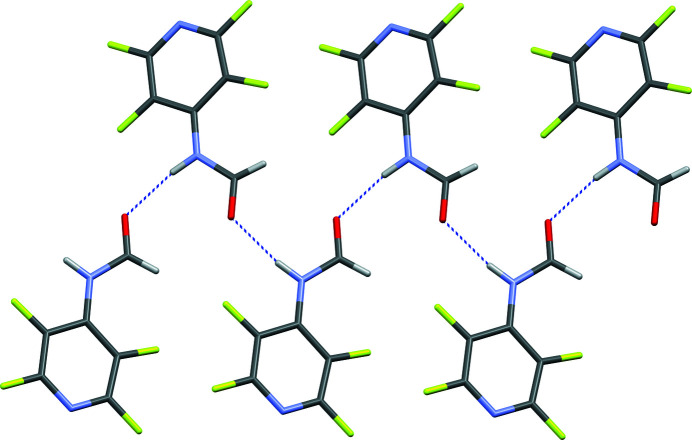
Inter­molecular hydrogen bonding forming a chain propagating along the *b*-axis direction where hydrogen bonds are represented with dashed lines.

**Figure 3:**
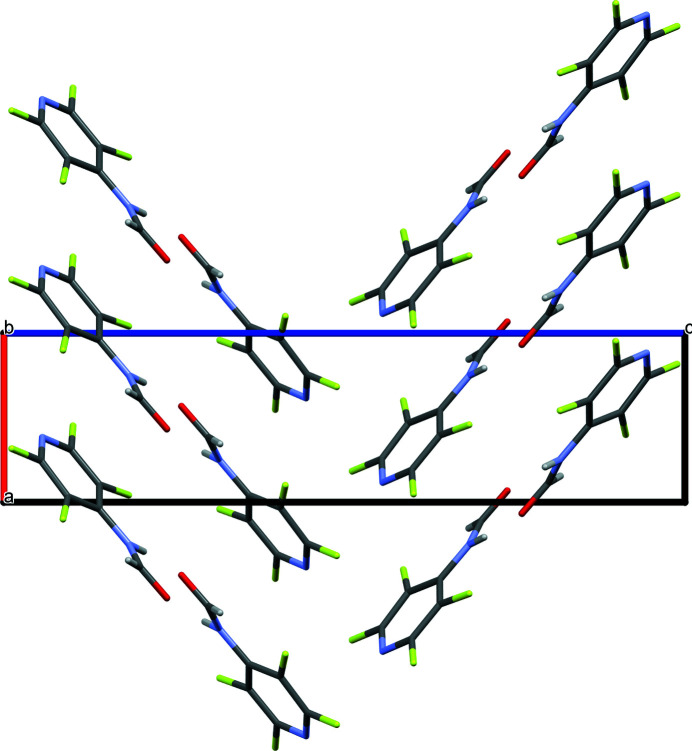
Extended structure as viewed looking down the *b*-axis showing the L-shape that is formed by the orthogonal pyridine rings of neighboring chains.

In the extended structure of (I) the mol­ecules are linked by N—H⋯O hydrogen bonds with a bond angle of 171 (3)° (Table 1 ▸), which suggests evidence of inter­mediate–strong hydrogen bonding (Arunan *et al.*, 2011 ▸). The hydrogen bonding generates chains of mol­ecules propagating along the *b*-axis direction in the extended structure (Fig. 2 ▸) with adjacent mol­ecules in the chain related by 2_1_ screw axis symmetry. Neighboring sets of chains form an L shape through a nearly orthogonal (84°) orientation of the pyridine rings in each chain (Fig. 3 ▸). This brings about short contacts between the pyridyl nitro­gen atoms and the π systems of these orthogonal pyridine rings (N⋯centroid = 3.502 Å; shortest N⋯C = 3.032 Å).

## Synthesis and crystallization

A 50 ml round-bottom flask was charged with 2,3,5,6-tetra­fluoro­pyridin-4-amine (0.1078 g, 0.6491mmol), *p*-toluene­sulfonic acid (0.0046 g, 0.027 mmol), trimethyl orthoformate (0.28 ml, 2.6 mmol), and toluene (5 ml). A Dean–Stark apparatus was filled with toluene (10 ml), and the solution was refluxed for 16 h. A homogenous colorless solution was obtained. Crystals were obtained by di­chloro­methane layered with hexa­nes, yielding orange needles. ^19^F{^1^H} NMR (CDCl_3_, δ): 91.1 (2F, *d*, –CF), 154.9 (2F, *d*, –CF). ^1^H NMR (CDCl_3_, δ): 9.00 (1H, *s*, –O=CH), 7.70 (1H, *s*, –NH).

## Refinement

Crystal data, data collection, and structure refinement details are summarized in Table 2 ▸.

## Supplementary Material

Crystal structure: contains datablock(s) I. DOI: 10.1107/S2414314622008045/hb4409sup1.cif

Structure factors: contains datablock(s) I. DOI: 10.1107/S2414314622008045/hb4409Isup2.hkl

Supporting information file. DOI: 10.1107/S2414314622008045/hb4409Isup3.cml

CCDC reference: 2196118

Additional supporting information:  crystallographic information; 3D view; checkCIF report

## Figures and Tables

**Table 1:** Hydrogen-bond geometry (Å, °)

*D*—H⋯*A*	*D*—H	H⋯*A*	*D*⋯*A*	*D*—H⋯*A*
N2—H2⋯O1^i^	0.87 (3)	1.96 (3)	2.814 (3)	171 (3)

Symmetry code: (i) 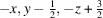 .

**Table 2:** Experimental details

Crystal data	
Chemical formula	C_6_H_2_F_4_N_2_O
*M*_r_	194.10
Crystal system, space group	Orthorhombic, *P*2_1_2_1_2_1_
Temperature (K)	100
*a*, *b*, *c* (Å)	5.1183 (4), 6.2707 (6), 20.6294 (16)
*V* (Å^3^)	662.11 (10)
*Z*	4
Radiation type	Mo *K*α
μ (mm^−1^)	0.21
Crystal size (mm)	0.28 × 0.06 × 0.05

Data collection	
Diffractometer	Bruker D8 Venture Photon 2
Absorption correction	Multi-scan (*SADABS*; Bruker, 2018 ▸)
*T*_min_, *T*_max_	0.897, 1.000
No. of measured, independent and observed [*I* > 2σ(*I*)] reflections	3556, 1309, 1200
*R*_int_	0.029
(sin θ/λ)_max_ (Å^−1^)	0.618

Refinement	
*R*[*F* ^2^ > 2σ(*F* ^2^)], *wR*(*F* ^2^), *S*	0.028, 0.066, 1.09
No. of reflections	1309
No. of parameters	122
H-atom treatment	H atoms treated by a mixture of independent and constrained refinement
Δρ_max_, Δρ_min_ (e Å^−3^)	0.14, −0.19
Absolute structure	Flack *x* determined using 424 quotients [(*I* ^+^)−(*I* ^−^)]/[(*I* ^+^)+(*I* ^−^)] (Parsons *et al.*, 2013 ▸)
Absolute structure parameter	−0.1 (6)

Computer programs: *APEX3* and *SAINT* (Bruker, 2018 ▸), *SHELXT2014/5* (Sheldrick, 2015*a* ▸), *SHELXL2016/6* (Sheldrick, 2015*b* ▸), and *SHELXTL* (Sheldrick, 2008 ▸).

## full crystallographic data

### Crystal data

**Table:**

C_6_H_2_F_4_N_2_O	*D*_x_ = 1.947 Mg m^−^^3^
*M**_r_* = 194.10	Mo *K*α radiation, λ = 0.71073 Å
Orthorhombic, *P*2_1_2_1_2_1_	Cell parameters from 2461 reflections
*a* = 5.1183 (4) Å	θ = 3.4–26.0°
*b* = 6.2707 (6) Å	µ = 0.21 mm^−^^1^
*c* = 20.6294 (16) Å	*T* = 100 K
*V* = 662.11 (10) Å^3^	Column, colourless
*Z* = 4	0.28 × 0.06 × 0.05 mm
*F*(000) = 384	

### Data collection

**Table:**

Bruker D8 Venture Photon 2 diffractometer	1200 reflections with *I* > 2σ(*I*)
Radiation source: Incoatec IµS	*R*_int_ = 0.029
φ and ω scans	θ_max_ = 26.0°, θ_min_ = 3.4°
Absorption correction: multi-scan (SADABS; Bruker, 2018)	*h* = −6→6
*T*_min_ = 0.897, *T*_max_ = 1.000	*k* = −7→7
3556 measured reflections	*l* = −25→25
1309 independent reflections	

### Refinement

**Table:**

Refinement on *F*^2^	Secondary atom site location: difference Fourier map
Least-squares matrix: full	Hydrogen site location: mixed
*R*[*F*^2^ > 2σ(*F*^2^)] = 0.028	H atoms treated by a mixture of independent and constrained refinement
*wR*(*F*^2^) = 0.066	*w* = 1/[σ^2^(*F*_o_^2^) + (0.0317*P*)^2^ + 0.041*P*] where *P* = (*F*_o_^2^ + 2*F*_c_^2^)/3
*S* = 1.09	(Δ/σ)_max_ < 0.001
1309 reflections	Δρ_max_ = 0.14 e Å^−^^3^
122 parameters	Δρ_min_ = −0.19 e Å^−^^3^
0 restraints	Absolute structure: Flack *x* determined using 424 quotients [(*I*^+^)-(*I*^-^)]/[(*I*^+^)+(*I*^-^)] (Parsons *et al.*, 2013)
Primary atom site location: dual	Absolute structure parameter: −0.1 (6)

### Special details

**Table:**

Geometry. All esds (except the esd in the dihedral angle between two l.s. planes) are estimated using the full covariance matrix. The cell esds are taken into account individually in the estimation of esds in distances, angles and torsion angles; correlations between esds in cell parameters are only used when they are defined by crystal symmetry. An approximate (isotropic) treatment of cell esds is used for estimating esds involving l.s. planes.

### Fractional atomic coordinates and isotropic or equivalent isotropic displacement parameters (Å^2^)

**Table:**

	*x*	*y*	*z*	*U*_iso_*/*U*_eq_	
F1	0.9516 (3)	−0.1033 (2)	0.60146 (7)	0.0280 (4)	
F2	0.5417 (3)	−0.0546 (2)	0.68314 (6)	0.0218 (4)	
F3	0.3999 (3)	0.6218 (2)	0.58674 (7)	0.0246 (4)	
F4	0.8168 (3)	0.5429 (2)	0.51143 (7)	0.0246 (4)	
O1	−0.0674 (4)	0.4673 (3)	0.73924 (8)	0.0239 (4)	
N1	0.8836 (4)	0.2207 (3)	0.55635 (10)	0.0194 (5)	
N2	0.2513 (4)	0.3050 (3)	0.68341 (10)	0.0176 (5)	
H2	0.208 (6)	0.193 (5)	0.7052 (15)	0.030 (8)*	
C1	0.8095 (5)	0.0761 (4)	0.59878 (12)	0.0186 (6)	
C2	0.6040 (5)	0.0998 (4)	0.64033 (11)	0.0172 (5)	
C3	0.4581 (5)	0.2879 (4)	0.64007 (11)	0.0153 (5)	
C4	0.5327 (5)	0.4385 (4)	0.59394 (11)	0.0168 (5)	
C5	0.7440 (5)	0.3963 (4)	0.55472 (11)	0.0188 (6)	
C6	0.1120 (5)	0.4818 (4)	0.70070 (12)	0.0195 (6)	
H6	0.155131	0.616615	0.682599	0.023*	

### Atomic displacement parameters (Å^2^)

**Table:**

	*U*^11^	*U*^22^	*U*^33^	*U*^12^	*U*^13^	*U*^23^
F1	0.0275 (9)	0.0267 (8)	0.0299 (8)	0.0124 (7)	0.0063 (8)	0.0033 (6)
F2	0.0255 (8)	0.0183 (7)	0.0215 (7)	0.0018 (7)	0.0038 (7)	0.0059 (6)
F3	0.0292 (9)	0.0193 (7)	0.0253 (8)	0.0059 (7)	0.0041 (8)	0.0053 (6)
F4	0.0272 (8)	0.0280 (8)	0.0187 (7)	−0.0051 (7)	0.0039 (7)	0.0064 (6)
O1	0.0232 (10)	0.0228 (9)	0.0255 (9)	0.0014 (9)	0.0100 (9)	−0.0038 (7)
N1	0.0152 (11)	0.0287 (11)	0.0143 (10)	−0.0001 (9)	−0.0007 (10)	−0.0007 (8)
N2	0.0183 (11)	0.0174 (10)	0.0170 (10)	−0.0002 (9)	0.0036 (11)	0.0011 (8)
C1	0.0167 (13)	0.0196 (12)	0.0195 (12)	0.0029 (10)	−0.0009 (12)	−0.0008 (10)
C2	0.0183 (13)	0.0185 (12)	0.0149 (12)	−0.0013 (10)	−0.0003 (12)	0.0013 (9)
C3	0.0146 (11)	0.0197 (11)	0.0117 (11)	−0.0002 (10)	−0.0011 (11)	−0.0029 (8)
C4	0.0182 (13)	0.0176 (11)	0.0146 (11)	0.0010 (11)	−0.0021 (11)	0.0005 (9)
C5	0.0211 (13)	0.0220 (12)	0.0132 (12)	−0.0055 (11)	−0.0026 (12)	0.0026 (10)
C6	0.0198 (14)	0.0183 (12)	0.0205 (12)	0.0005 (11)	0.0016 (13)	−0.0021 (9)

### Geometric parameters (Å, º)

**Table:**

F1—C1	1.341 (3)	N2—C3	1.390 (3)
F2—C2	1.348 (3)	N2—H2	0.87 (3)
F3—C4	1.343 (3)	C1—C2	1.365 (3)
F4—C5	1.335 (3)	C2—C3	1.396 (3)
O1—C6	1.218 (3)	C3—C4	1.394 (3)
N1—C5	1.313 (3)	C4—C5	1.376 (4)
N1—C1	1.316 (3)	C6—H6	0.9500
N2—C6	1.366 (3)		
			
C5—N1—C1	116.0 (2)	N2—C3—C2	118.0 (2)
C6—N2—C3	128.9 (2)	C4—C3—C2	115.4 (2)
C6—N2—H2	113 (2)	F3—C4—C5	119.8 (2)
C3—N2—H2	118 (2)	F3—C4—C3	121.1 (2)
N1—C1—F1	116.7 (2)	C5—C4—C3	119.1 (2)
N1—C1—C2	124.4 (2)	N1—C5—F4	116.3 (2)
F1—C1—C2	118.9 (2)	N1—C5—C4	125.0 (2)
F2—C2—C1	121.0 (2)	F4—C5—C4	118.7 (2)
F2—C2—C3	118.9 (2)	O1—C6—N2	120.2 (2)
C1—C2—C3	120.1 (2)	O1—C6—H6	119.9
N2—C3—C4	126.5 (2)	N2—C6—H6	119.9
			
C5—N1—C1—F1	−179.4 (2)	N2—C3—C4—F3	−1.1 (4)
C5—N1—C1—C2	−0.8 (4)	C2—C3—C4—F3	176.8 (2)
N1—C1—C2—F2	−179.1 (2)	N2—C3—C4—C5	179.8 (2)
F1—C1—C2—F2	−0.6 (3)	C2—C3—C4—C5	−2.3 (3)
N1—C1—C2—C3	−0.7 (4)	C1—N1—C5—F4	−179.5 (2)
F1—C1—C2—C3	177.9 (2)	C1—N1—C5—C4	0.7 (4)
C6—N2—C3—C4	−15.2 (4)	F3—C4—C5—N1	−178.2 (2)
C6—N2—C3—C2	167.0 (2)	C3—C4—C5—N1	0.9 (4)
F2—C2—C3—N2	−1.3 (3)	F3—C4—C5—F4	2.0 (3)
C1—C2—C3—N2	−179.8 (2)	C3—C4—C5—F4	−178.9 (2)
F2—C2—C3—C4	−179.3 (2)	C3—N2—C6—O1	179.0 (2)
C1—C2—C3—C4	2.2 (3)		

### Hydrogen-bond geometry (Å, º)

**Table:**

*D*—H···*A*	*D*—H	H···*A*	*D*···*A*	*D*—H···*A*
N2—H2···O1^i^	0.87 (3)	1.96 (3)	2.814 (3)	171 (3)

Symmetry code: (i) −*x*, *y*−1/2, −*z*+3/2.
